# Recurrence of Primary Pulmonary Meningioma 10 Years after Surgery: A Case Report

**DOI:** 10.70352/scrj.cr.25-0083

**Published:** 2025-04-16

**Authors:** Ting Fang, Haibo Huang

**Affiliations:** 1 Shandong Second Medical University, Weifang, Shandong, China; 2 Department of Thoracic Surgery, Yantai Yuhuangding Hospital, Medical College of Qingdao University, Yantai, Shandong, China

**Keywords:** primary pulmonary meningioma, recurrence, surgery

## Abstract

**INTRODUCTION:**

Primary pulmonary meningioma (PPM) is an exceptionally rare form of ectopic meningioma, with few cases documented in the literature, and even fewer reports of recurrence following surgical resection.

**CASE PRESENTATION:**

This case study details a 69-year-old male patient diagnosed with PPM postoperatively, who experienced a recurrence 10 years after the initial surgery, necessitating a second surgical intervention. The postoperative pathological diagnoses from both surgeries confirmed transitional type primary pulmonary meningioma. The Ki-67 index from the first surgery was less than 1%, while the second postoperative pathology demonstrated a Ki-67 index of 5%.

**CONCLUSION:**

The case emphasizes that, despite its benign classification, PPM has a potential for recurrence, underscoring the importance of ongoing, long-term follow-up in post-surgical management.

## INTRODUCTION

Primary pulmonary meningioma (PPM) is an exceptionally rare form of ectopic meningioma, with only sporadic cases documented in the literature and fewer reports of recurrence following resection. This study presents a unique case of PPM that recurred 10 years after initial surgical resection.

## CASE PRESENTATION

**Fig. 1:**
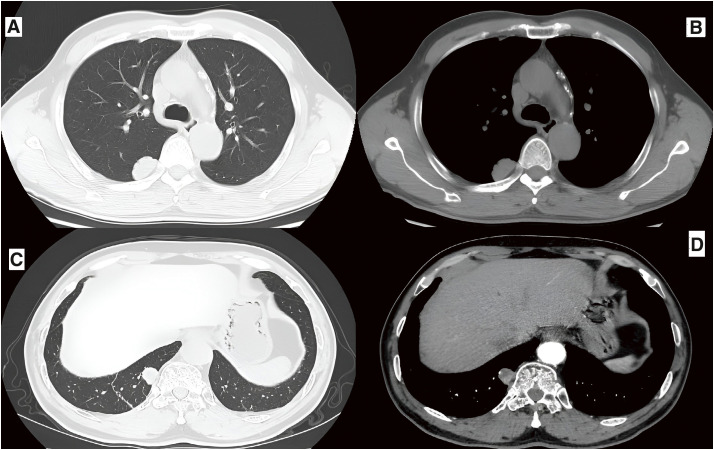
(**A**) Lung window of the chest CT prior to the patient’s initial surgery, revealing a lesion in the dorsal segment of the right lower lobe, measuring approximately 3 cm in maximum diameter, with subtle lobulation and broad-based adhesion to the adjacent pleura. (**B**) Mediastinal window of the same preoperative CT showing homogeneous lesion density without calcification, displaying a CT attenuation value of approximately 33 Hounsfield Units (HU). (**C**) Lung window of the chest CT before the second surgery, illustrating a lesion in the posterior basal segment of the right lower lobe, measuring approximately 2 cm in maximum diameter, with mild lobulation and no significant spiculation. (**D**) Mediastinal window of the second preoperative CT demonstrating homogeneous density on contrast-enhanced scan, exhibiting mild heterogeneous enhancement.

**Fig. 2:**
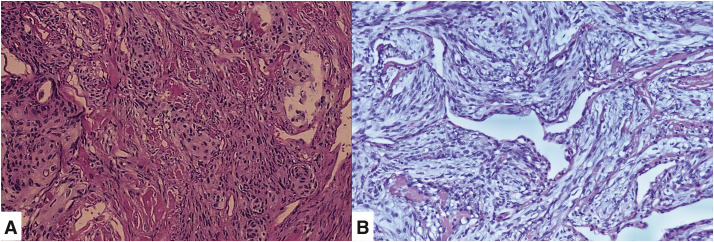
Hematoxylin and eosin (H&E)-stained pathological sections (magnification ×200) (**A**) from the first surgery and the (**B**) second surgery, demonstrating a spindle cell neoplasm characterized by fascicular, whorling, and fusiform patterns of tumor cell.

This case report presents a 69-year-old male patient initially admitted in March 2014 following detection of a lung mass during routine health screening. Computed tomography (CT) of chest revealed a well-defined, lobulated mass in the posterior segment of the right upper lobe, measuring 2.8 × 1.7 cm, with uniform density (CT value: 33 HU) and a broad pleural base (**Fig. 1A**). Brain magnetic resonance imaging (MRI) showed no abnormalities. Thoracoscopic wedge resection was performed, and postoperative pathology confirmed a diagnosis of pulmonary multifocal ectopic meningioma (transitional type) involving the visceral pleura (**Fig. 2A**). Immunohistochemistry demonstrated tumor positivity for vimentin, epithelial membrane antigen (EMA), progesterone receptor (PgR), and thyroid transcription factor-1 (TTF-1) and negativity for cytokeratin and B-cell lymphoma-2 (Bcl-2), with a Ki-67 proliferation index of <1%. The patient recovered well postoperatively.

During regular follow-up, annual chest CT scans remained normal for 8 years. However, 2 years ago, a new nodule appeared in the right lower lobe and exhibited gradual enlargement. Upon readmission, CT identified the nodule in the posterior basal segment of the right lower lobe, measuring 1.6 × 1.9 cm (**Fig. 1B**), while brain MRI remained normal. A second thoracoscopic wedge resection was performed, and postoperative pathology identified primary pulmonary meningioma (transitional type) involving the visceral pleura (**Fig. 2B**), with increased cellularity and focal cellular activity. Immunohistochemically, the tumor was positive for vimentin, EMA, PgR, TTF-1, and somatostatin receptors-2, and negative for cytokeratin, Bcl-2, CD34, and S-100, with a Ki-67 index of approximately 5%. This case highlights the potential for recurrence in pulmonary ectopic meningioma and underscores the importance of long-term monitoring.

## DISCUSSION

Primary ectopic meningioma is an exceptionally rare tumor, typically occurring in the head and neck regions but also observed in the lungs, mediastinum, and retroperitoneum.^1)^ Given that the lungs are a frequent site for metastatic meningiomas, a diagnosis of PPM must be approached cautiously, with intracranial and intraspinal metastasis thoroughly excluded. Imaging plays a critical role in differentiating PPM from primary central nervous system meningiomas, thereby confirming the diagnosis.^2,3)^

While the pathogenesis of PPM remains unclear, it is hypothesized that ectopic meningiomas may originate from pluripotent subpleural mesenchymal cells or ectopic embryonic remnants of arachnoid cells. This case report provides insights into the diagnostic considerations and potential origins of PPM.^4)^

PPMs exhibit imaging characteristics that aid in distinguishing them from other pulmonary tumors. Typically, PPMs appear on chest CT scans as isolated, round, solid, and well-defined nodules or masses, occasionally with calcification. Less commonly, PPMs may present as ground-glass nodules or multiple cystic lesions. Differential diagnoses include pulmonary hamartoma, sclerosing pneumocytoma, and carcinoid tumors. The literature indicates that benign PPMs generally range from 0.4 to 6 cm in diameter (median: 2 cm), while malignant PPMs range from 1.5 to 15 cm (median: 6.4 cm).^5)^ However, tumor size alone is not sufficient to distinguish benign from malignant PPMs. In this case, histopathological analysis of the resected specimens from both surgical interventions revealed tumor dimensions of 3 and 2 cm, respectively, with visceral pleural involvement, a feature indicative of malignant potential. Although PPMs may show F-fluorodeoxyglucose (FDG) uptake, positron emission tomography (PET)-CT is not typically advantageous in their diagnosis.^3,4)^

Pathologically, PPMs are classified into three principal histological subtypes: meningothelial, fibroblastic, and transitional. Of these, the transitional variant demonstrates the highest prevalence, whereas the meningothelial subtype exhibits the greatest rarity. Histopathologically, PPMs are composed of fusiform, polygonal, or ovoid cells arranged in lobules or spirals, usually with no mitosis. Immunohistochemical analysis frequently shows positive for markers such as vimentin, EMA, cytokeratin, desmin, and S-100, findings consistent with this case. The Ki-67 proliferation index is considered a prognostic marker; a rate below 3% correlates with a low likelihood of recurrence. Most PPMs are benign, with low rates of recurrence or distant metastasis.^5–9)^ In this case, the initial Ki-67 index was below 1%, while the subsequent pathology reported approximately 5%, indicating increased proliferative activity. The case highlights that while benign PPMs generally have favorable outcomes, ongoing monitoring is necessary due to the potential for progression or recurrence.

Complete surgical resection remains an effective treatment for this disease. While PPMs typically follow a slow course with many patients remaining disease-free for up to 24 years, recurrence is infrequent but has been documented. The potential for malignancy in PPMs varies, with karyotype influencing the tumor’s behavior. Prayson had described a case of PPM with both local and distant recurrence within 5 months of initial excision.^5)^

## CONCLUSION

In this case, a PPM was diagnosed 10 years after initial resection, with recurrence identified during follow-up. Pathological analysis of the recurrent tumor indicated benign features, and metastasis was not considered. Both tumors were completely resected, and the patient remains under close observation. This case highlights that even benign PPMs are at risk of recurrence, emphasizing the importance of long-term follow-up. Although there is no established treatment for this rare condition, complete tumor excision remains the cornerstone of management.

## ACKNOWLEDGMENTS

The authors extend their sincere gratitude to Dr. Zhiqiang Lang of the Department of Pathology in their hospital for his expert guidance on the pathological aspects of this article.

## DECLARATIONS

### Funding

None.

### Authors’ contributions

TF drafted the work.

HH revised it.

All authors contributed to manuscript writing and editing.

All authors have read and approved the final version of this manuscript.

### Availability of data and materials

Data sharing is not applicable to this article as no datasets were generated or analyzed during the current study.

### Ethics approval and consent to participate

Ethical approval for this case report was waived by the institutional Review Board of Yantai YuHuang Ding Hospital, as it involved retrospective analysis of anonymized clinical records. Written informed consent was obtained from the patient for both participation in the study and publication of anonymized data.

### Consent for publication

This patient signed an informed consent approved by the institutional Review Board of the hospital. A copy of the written consent is retained by the corresponding author at the institution and secured per hospital protocol; this document can be accessed by the corresponding author.

### Competing interests

The authors declare that they have no competing interests.
